# Intrinsic anomalous, spin and valley Hall effects in ’ex-so-tic’ van-der-Waals structures

**DOI:** 10.1038/s41598-024-74596-x

**Published:** 2024-10-11

**Authors:** I. Wojciechowska, A. Dyrdał

**Affiliations:** Faculty of Physics and Astronomy, ISQI, Adam Mickiewicz University in Poznań, ul. Uniwersytetu Poznańskiego 2, 61-614 Poznań, Poland

**Keywords:** Electronic properties and materials, Spintronics, Surfaces, interfaces and thin films

## Abstract

We consider the anomalous, spin, valley, and valley spin Hall effects in a pristine graphene-based van-der-Waals (vdW) heterostructure consisting of a bilayer graphene (BLG) sandwiched between a semiconducting van-der-Waals material with strong spin-orbit coupling (e.g., $$\hbox {WS}_2$$) and a ferromagnetic insulating vdW material (e.g. $$\hbox {Cr}_2$$$$\hbox {Ge}_2$$$$\hbox {Te}_6$$). Due to the exchange proximity effect from one side and spin-orbit proximity effect from the other side of graphene, such a structure is referred to as graphene based ’ex-so-tic’ structure. First, we derive an effective Hamiltonian describing the low-energy states of the structure. Then, using the Green’s function formalism, we obtain analytical results for the Hall conductivities as a function of the Fermi energy and gate voltage. For specific values of these parameters, we find a quantized valley Hall conductivity.

## Introduction

Two-dimensional (2D) van-der-Waals (vdW) materials, being promising materials for further development of spintronics and a real step towards further miniaturization of electronic devices^1,2^, attract currently an enormous attention. The most fascinating aspect of van-der-Waals structures is the possibility of designing their electronic, magnetic and topological properties on demand. Such a possibility is a consequence of stacking selected 2D van-der-Waals crystals (having specific physical properties) and proximity effects emerging in these stacks^3^. Accordingly, van-der-Waals hybrid structures, created by stacking 2D crystals that reveal a wide range of physical properties and phases (e.g., insulators, semiconductors, metals, superconductors, magnetics, ferroelectrics, etc.^4–8^), constitute a unique class of materials with a combination of different physical properties, that can be tuned not only by external fields and forces, but also due to mutual coupling between different phases of matter within the structure. This is the case, for example, in van-der-Waals multiferroics^9,10^, that can be obtained by stacking two van-der-Waals crystals: ferroelectric and ferromagnetic ones.

According to the main paradigm of spin-electronics, which assumes the usage of electron spin on equal footing with its charge, the all-electrical control of the spin degree of freedom and search for additional degrees of freedom that can couple to the spin are of special interest^11^. Naturally, the spin-orbit-driven transport phenomena, such as current-induced spin polarization, anomalous and spin Hall effects and their quantum counterparts^1,12–14^ have become a hallmark of modern spin electronics. Importantly, van-der-Waals hybrid structures with their additional degrees of freedom, such as the valley or pseudospin (related to the sublattices or other orbital properties of vdW structures), seem to be a perfect platform for designing spintronic devices of a new generation^6,7,15–18^. Among the van-der-Waals hybrid structures, the stacks containing graphene are of great interest. In this particular case, one can design structures that exploit the unique and high-quality electronic properties of graphene, enriched with additional physical properties revealed as a consequence of the proximity effects due to its contact with adjacent layers, see for example ^19–34^ and reviews ^35–40^.

**Fig. 1:**
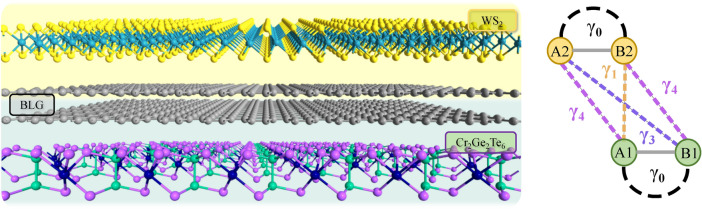
’Ex-so-tic’ van-der Waals structure (on the left) consisting of a bilayer graphene (BLG) between other 2D vdW crystals: semiconducting with strong SOC (e.g., transition metal dichalcogenide such as $$\hbox {WS}_2$$) and ferromagnetic insulator such as $$\hbox {Cr}_2$$$$\hbox {Ge}_2$$$$\hbox {Te}_6$$. The intralayer hopping parameter $$\gamma _{0}$$ and interlayer hopping parameters $$\gamma _{1,3,4}$$, taken into account in the considered Hamiltonian of BLG, are indicated on the right side. Here A1,2 (B1,2) correspond to the graphene top (1) or bottom (2) layer, and graphene sublattice A or B.

Here we consider the Hall effects in a pristine ’ex-so-tic’ graphene-based van-der-Waals heterostructure^24^. In other words, we restrict our considerations to the intrinsic transport properties. Accordingly we do not consider the extrinsic scattering mechanisms leading to the Hall effects, such as skew-scattering or side-jump mechanisms^12^ that may be due to spin-orbital scatterers or interplay between Rashba and exchange (or valley-Zeeman) coupling in the presence of scalar impurities^41,42^. The name ’ex-so-tic’ structure originates from the building blocks in the structure under consideration. Namely, a bilayer of graphene (BLG) is deposited on a magnetic 2D insulator, e.g., on ${\mathrm {Cr_{2}Ge_{2}Te_{6}}}$ (CGT), that creates a strong proximity-induced exchange field in the bottom (1st) layer of BLG and only weakly affects the electronic properties of the top (2nd) layer. Additionally, from the top, the BLG is covered by a semiconducting 2D transition metal dichalcogenide (TMDC), i.e. ${\mathrm {WS_2}}$, responsible for a strong proximity-induced spin-orbit coupling in the top layer of BLG and a weak one in the bottom layer. The structure is presented in Fig 1. Interestingly, the electronic band structure is highly tuneable by an external gate voltage, that dramatically changes the impact of the spin-orbit and exchange interactions on the electronic conduction and valence bands (for details, see^24^).

Using an effective Hamiltonian describing the low-energy bands in the vicinity of the charge neutrality point, we analyse in detail the topological nature of the ’ex-so-tic’ structure and its various Hall characteristics. Accordingly, our paper provides a broad basis on how to understand the topology of the structure from simple measurements of various Hall effects.

## Model of ’ex-so-tic’ structure and its topological properties

We consider the ’ex-so-tic’ graphene-based van-der-Waals heterostructure, i.e. the system consisting of a bilayer of graphene (BLG) deposited on the magnetic 2D insulator, ${\mathrm {Cr_{2}Ge_{2}Te_{6}}}$ (CGT), that creates a strong proximity-induced exchange field in the bottom layer of BLG, and covered by a semiconducting 2D TMDC, ${\mathrm {WS_2}}$, responsible for a strong proximity spin-orbit coupling in the top layer of BLG. The structure is presented schematically in Fig 1.

**Fig. 2:**
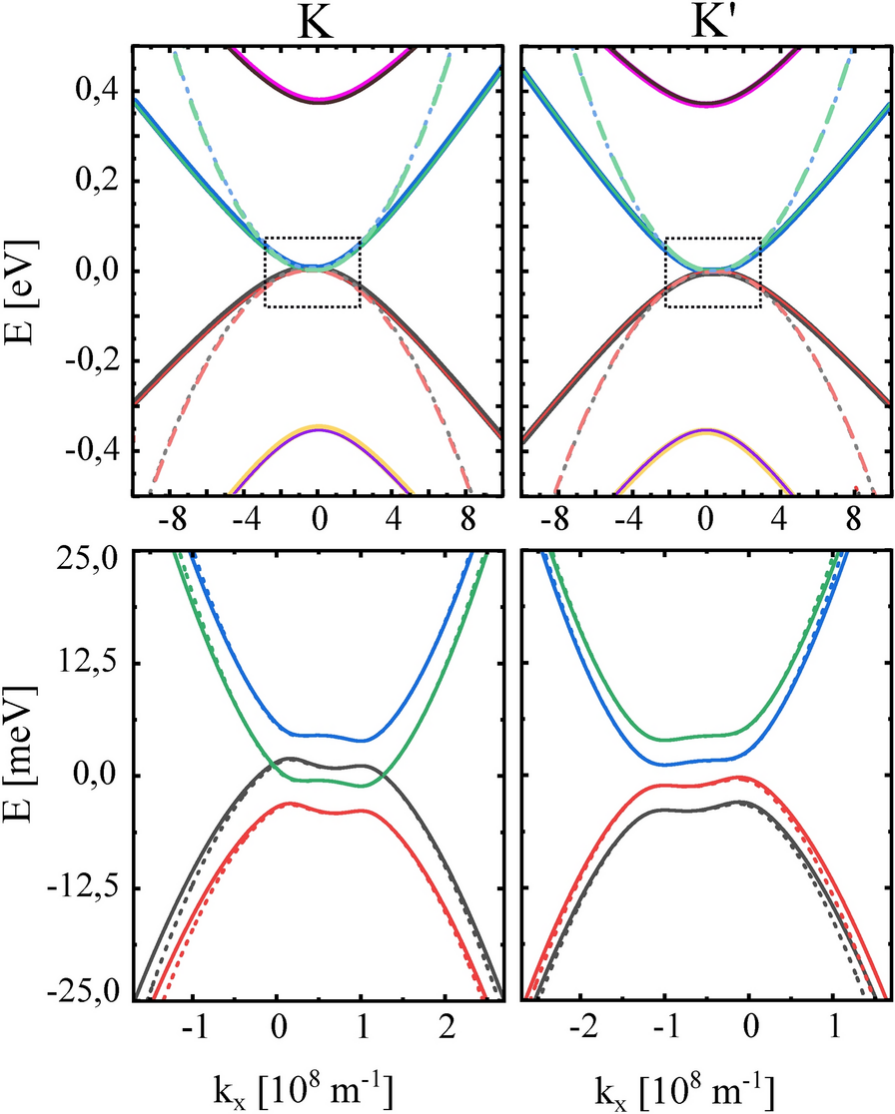
Top panel: Energy spectrum around the K and K’ points, obtained based on the ${\textbf{k}}\cdot {\textbf{p}}$ model described by the 8x8 ${\textbf{k}}\cdot {\textbf{p}}$ Hamiltonian (solid lines) and by the reduced Hamiltonian (dashed lines). Bottom panel: The low-energy states around the K and K’ points, where the eigenvalues of ${\textbf{k}}\cdot {\textbf{p}}$ Hamiltonian (solid lines) are compared with the eigenvalues of the low-energy reduced Hamiltonian given by Eq.(1) (dotted lines). From these energy spectra follows that the reduced Hamiltonian is a good approximation of the $\textbf{k}\cdot \textbf{p}$ model in the energy window between -25meV and +25meV. The band structures are plotted for the following parameters: $\gamma _{0} = 2.432$ eV, $\gamma _{1} = 0.365$ eV, $\gamma _{3} = -0.273$ eV, $\gamma _{4} = -0.164$ eV, $\lambda _{I}^{A1} = \lambda _{I}^{B1} = 0$, $\lambda _{I}^{A2} = 1.132$ meV, $\lambda _{I}^{B2} = -\lambda _{I}^{A2}$, $\lambda _{EX}^{A1} = \lambda _{EX}^{B1} = -3.874$ meV, $\lambda _{EX}^{A2} = \lambda _{EX}^{B2} = 0$, $\Delta = 8.854$ meV, a = 2.5

The electronic structure of the bilayer graphene, proximitized to the 2D semiconducting TMDC from one side and to the 2D ferromagnetic insulator from the other side, was calculated numerically within the DFT methods by Zollner et al.^24^. By fitting the full energy spectrum to the effective 8*x*8 kp-Hamiltonian, the authors obtained the relevant parameters describing the kp-Hamiltonian. The corresponding band structure consists of four pairs of subbands (for each K point): two pairs form the valence and conduction bands close to the Fermi level, and two other pairs correspond to the valence and conduction bands shifted in energy away from the Fermi level by about $\pm 0.36$eV. The latter conduction and valence bands are formed mainly from the $p_z$ orbitals at atoms *A*1 and *B*2, whereas the former bands are formed mainly from the orbitals of atoms *A*2 and *B*1. The full spectrum described by the the effective 8*x*8 kp-Hamiltonian is shown by the solid lines in the top panel of Figure 2.

As the transport properties at low temperatures are determined by the four low-energy bands (for each Dirac point) in the vicinity of the Fermi level, for the purpose of transport calculations we developed the reduced Hamiltonian, that describes the physics related only to the four bands located in the vicinity of the charge neutrality point. This Hamiltonian takes the form (see the Supplementary material for its derivation):

1 $$\begin{aligned} \begin{aligned} {\hat{H}}^{\scriptscriptstyle {\tau }}_{{\scriptscriptstyle {B_{1}A_{2}}}} =&-\frac{v^2}{\gamma _{1}} \left( 1+ \gamma _{40}^{2} \right) \left( (k_x^2 - k_y^2) \hat{\eta }_{x} - 2 k_x k_y \hat{\eta }_{y}\right) \otimes {\hat{s}}_{0} - 2 \frac{v^2}{\gamma _{1}} \gamma _{40} k^{2} (\hat{\eta }_{0}\otimes {\hat{s}}_{0}) - v \gamma _{30} (\tau k_{\scriptscriptstyle {x}}\hat{\eta }_{x} + k_{\scriptscriptstyle {y}}\hat{\eta }_{y}) \otimes {\hat{s}}_{0} \\&- \lambda _{\scriptscriptstyle {EX}} (\hat{\eta }_{+} \otimes {\hat{s}}_{z}) + \lambda _{\scriptscriptstyle {I}}\tau (\hat{\eta }_{-} \otimes {\hat{s}}_{z} ) + V (\hat{\eta }_{z} \otimes {\hat{s}}_{0} ), \end{aligned} \end{aligned}$$

where $$v = \hbar v_{F}$$ ($$\hbar$$ - modified Planck constant, $$v_{F}$$ - Fermi velocity), $$\gamma _{0,1,3,4}$$ define the intralayer electron hopping between nearest neighbors, as well as interlayer hoppings between nearest and next nearest sites, as indicated in Fig.1, *a* is the lattice constant of graphene, *V* describes the effect of gate voltage (transverse displacement field), and $$\Delta$$ is the so-called orbital gap, being a consequence of the asymmetry in the energy shift of the bonding and antibonding states. The proximity exchange coupling of the bottom (1st) layer is described by the parameter $$\lambda _{EX} = \lambda _{EX}^{A1} = \lambda _{EX}^{B1}$$, whereas the proximity-induced spin-orbit coupling of the Valley-Zeeman type in the top (2nd) layer is defined by the parameter $$\lambda _{I} = \lambda _{I}^{A2} = - \lambda _{I}^{B2}$$. The matrices $$\hat{\eta }_{\alpha }$$,$$\hat{\eta }_{0}$$ represent Pauli matrices and the identity matrix acting in the B1-A2 dimer space, $$k_{\scriptscriptstyle {\pm }} = k_{\scriptscriptstyle {x}} \tau \pm ik_{\scriptscriptstyle {y}}$$, and $$\hat{\eta }_{\pm } =$$$$\frac{1}{2}(\sigma _{z} \pm \sigma _{0})$$. The derivation of this reduced Hamiltonian is based on the Green’s function method^35,43^, and is presented in the supplementary material. In graphene-based van-der-Waals hybrid structures, the Rashba spin-orbit coupling is usually present and can be tuned by the twist of graphene with respect to the substrate or by gating^25,44–49^. However, according to the DFT data for $$\hbox {Cr}_2$$$$\hbox {Ge}_2$$$$\hbox {Te}_6$$/BLG/$$\hbox {WS}_2$$ published in Ref.^24^, the spin-polarization of electrons related to the considered electronic bands is predominantly out-of-plane. This indicates that the Rashba coupling has a relatively small impact on the electronic structure. In fact, the first principles calculations of Ref. ^24^ find that the Rashba parameter entering the effective model can be safely set to zero. For simplicity, we neglect the effect of Rashba SOC in what follows. Moreover, to be consistent with Ref. ^24^, for the purpose of our study, we decided to use parameters describing the above Hamiltonian as derived in Ref.^24^ for this particular ’ex-so-tic’ heterostructure. However, one should note that some recent experiments indicate that spin-orbit coupling parameters derived based on first-principle methods are underestimated and realistic values of proximity-induced SOC can be remarkably larger (see for example Refs. ^48,49^).

The eigenvalues of Hamiltonian (1) take the form:

2 $$\begin{aligned} & E_{1,2}^\tau = - F^{\tau }_{{\textbf{k}}} \pm \frac{1}{2}(\lambda _{\scriptscriptstyle {EX}} - \tau \lambda _{\scriptscriptstyle {I}}) - 2 \frac{\gamma _{40}}{\gamma _{1}} v^{2}k^{2} \end{aligned}$$

3 $$\begin{aligned} & E_{3,4}^\tau = F^{\tau }_{{\textbf{k}}} \pm \frac{1}{2}(\lambda _{\scriptscriptstyle {EX}}-\tau \lambda _{\scriptscriptstyle {I}}) - 2 \frac{\gamma _{40}}{\gamma _{1}} v^{2}k^{2} \hspace{0.3cm} \end{aligned}$$

where

4 $$\begin{aligned} F^{\tau }_{{\textbf{k}}}= \left[ v^{2} \gamma _{30}^{2} k^{2} +\frac{v^{4}}{\gamma _{1}^{2}} (1 + \gamma _{40}^{2})^{2} k^{4} + \frac{1}{4}(2V\pm (\lambda _{\scriptscriptstyle {EX}} + \tau \lambda _{\scriptscriptstyle {I}}))^{2} - \frac{\tau }{2} \gamma _{1}\gamma _{3}(1+\gamma _{40}^{2}) k_{x}(k_{x}^{2}-3 k_{y}^{2}) \right] ^{1/2} . \end{aligned}$$

The energy spectrum related to the reduced Hamiltonian is presented in Fig. 2 by the dashed lines (in both panels). From this figure follows that in the energy window between -25meV and +25meV, the reduced model is a good approximation of the $\textbf{k}\cdot \textbf{p}$ model.

**Fig. 3:**
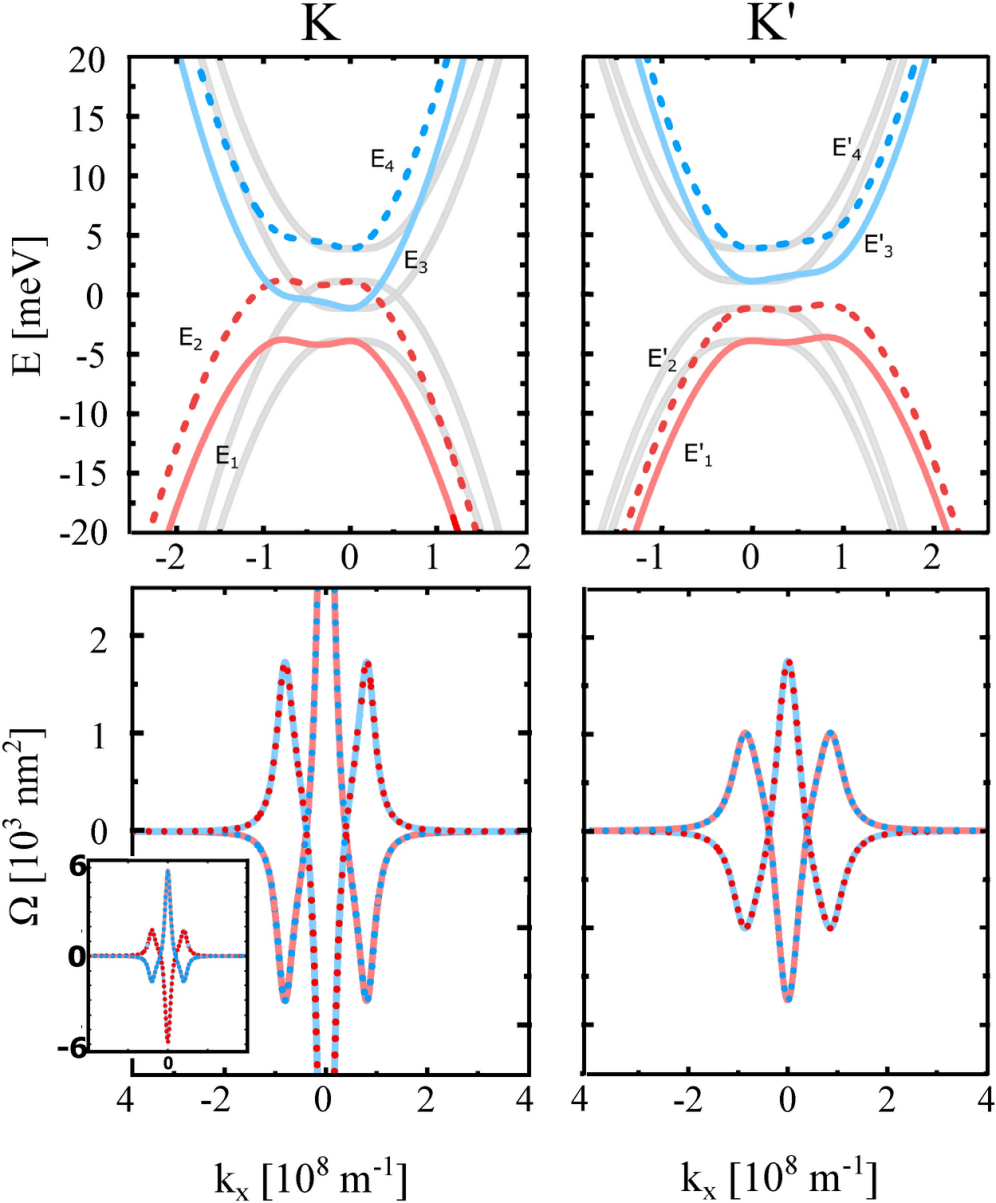
Electronic band structure corresponding to the reduced Hamiltonian (1) describing ’ex-so-tic’ vdW structure (top panel). Red and blue curves present the eigenvalues given by Eqs. (2) and (3). For comparison, the band structure for $\gamma _{3,4} = 0$ is plotted in grey. The bottom panel presents the Berry curvature corresponding to the bands presented in the top panel. The parameters are as in Fig. 2.

The topological properties of the electronic structure are described by the Berry curvature:

5 $$\begin{aligned} {\varvec{\Omega }}_{j} = \nabla _{\varvec{k}} \times \varvec{A}_{j}(\varvec{k}), \end{aligned}$$

where $\varvec{A}_{j} = i \langle \psi _{j} | \nabla _{\varvec{k}} | \psi _{j} \rangle$ is the Berry connection. The eigenvalues of the reduced Hamiltonian are shown in Fig.3 (red and blue lines in the top panel) for zero gate voltage, while the corresponding Berry curvatures are shown in the bottom panel. However, for calculating transport characteristic, we make some additional simplifications. Accordingly, we note that the effects of interlayer hoppings between the sites A1-A2 and B1-B2, defined by $\gamma _{4}$, and of the hoppings between the sites A2-B1, defined by $\gamma _{3}$ (see Fig. 1), are small and only lead to small corrections in the relevant electronic states. In fact, the hoppings $\gamma _{3,4}$ introduce a small tilting of the energy branches, as well as their weak shift from the K/K’ points, as depicted in Fig.3 (top panel), where the solid gray lines denote the eigenvalues (2) and (3) for $\gamma _{3,4} = 0$. In the context of transport characteristics studied in this manuscript, the assumption $\gamma _{3,4} = 0$ does not affect general trends but only leads to small quantitative changes. In turn, a big advantage of such a simplification is the possibility of obtaining analytical formulas for transport characteristics.

The eigenvalues of Hamiltonian (1) for the case ${\gamma _{3,4}=0}$ take the simple forms:

6 $$\begin{aligned} & E_{1,2}^\tau = -\sqrt{\frac{v^{4}}{\gamma _{1}^{2}} k^{4} + \left( V\pm \frac{1}{2}(\lambda _{\scriptscriptstyle {EX}} + \tau \lambda _{\scriptscriptstyle {I}})\right) ^{2}} \pm \frac{1}{2}(\lambda _{\scriptscriptstyle {EX}} - \tau \lambda _{\scriptscriptstyle {I}}), \end{aligned}$$

7 $$\begin{aligned} & E_{3,4}^\tau = \sqrt{\frac{v^{4}}{\gamma _{1}^{2}} k^{4} +\left( V\pm \frac{1}{2}(\lambda _{\scriptscriptstyle {EX}} + \tau \lambda _{\scriptscriptstyle {I}})\right) ^{2}} \pm \frac{1}{2}(\lambda _{\scriptscriptstyle {EX}} - \tau \lambda _{\scriptscriptstyle {I}}). \end{aligned}$$

In turn, Berry curvatures for the valence bands $E^{\tau }_{1,2}$ read:

8 $$\begin{aligned} \Omega _{1,2}^{\tau } =\tau \frac{4\pi \frac{v^{4}}{\gamma _{1}^{2}} k^{2} \left( V\pm \frac{1}{2}(\lambda _{\scriptscriptstyle {EX}} + \tau \lambda _{\scriptscriptstyle {I}})\right) }{ \left( \frac{v^{4}}{\gamma _{1}^{2}} k^{4} + \left( V\pm \frac{1}{2}(\lambda _{\scriptscriptstyle {EX}} + \tau \lambda _{\scriptscriptstyle {I}}) \right) ^{2} \right) ^{3/2}}, \end{aligned}$$

where $\tau = \pm 1$ indicates the valley K/K’, respectively, and the signs ± correspond respectively to the bands $E^\tau _{1,2}$. In turn, Berry curvatures for the conduction bands $E^{\tau }_{3,4}$ are:

9 $$\begin{aligned} \Omega _{3}^{\tau } = - \Omega _{1}^{\tau } \qquad \Omega _{4}^{\tau } = - \Omega _{2}^{\tau }. \end{aligned}$$

**Fig. 4:**
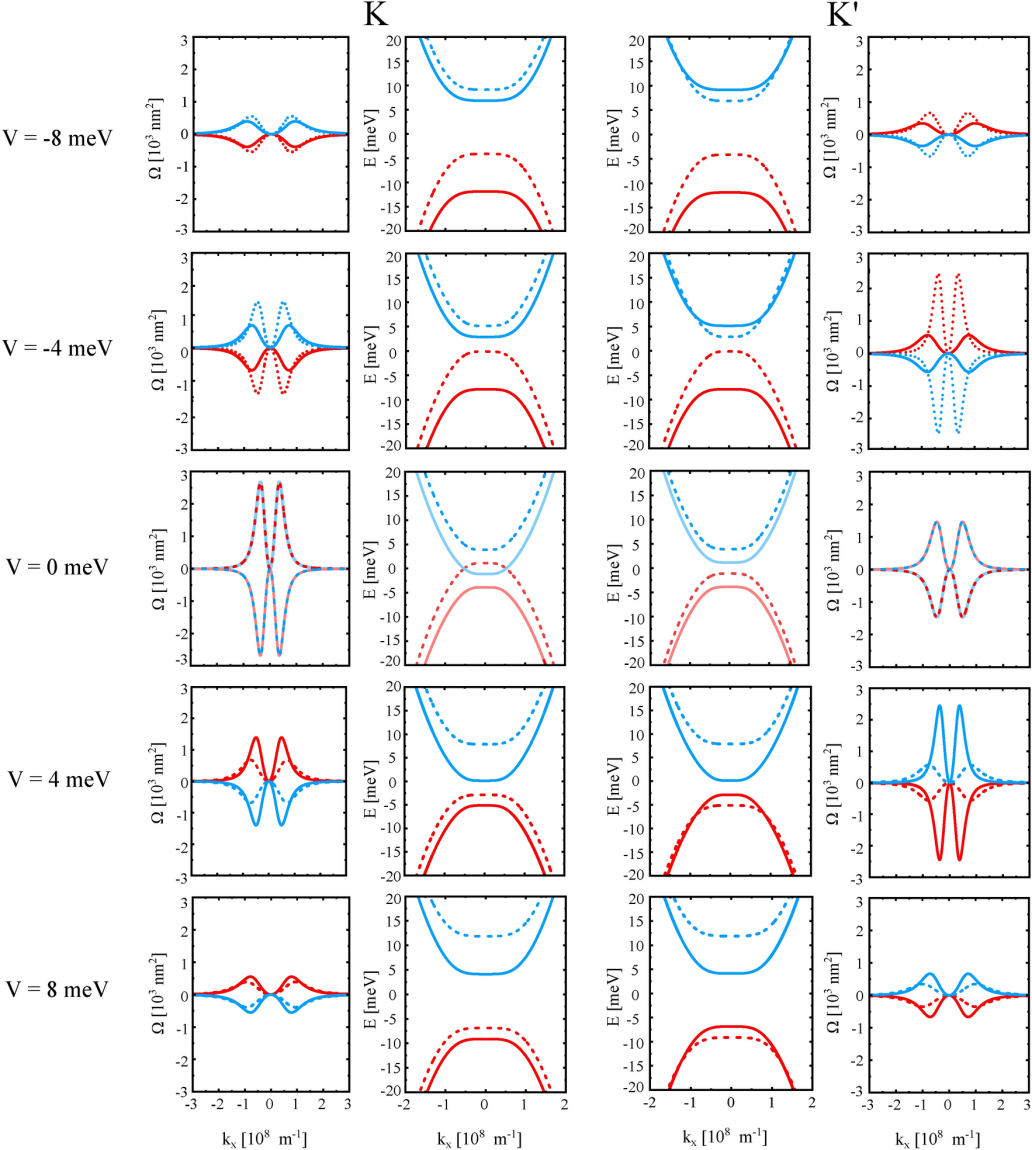
Band structure and the corresponding Berry curvature of ’ex-so-tic’ structure at K and K’ point for indicated values of the gate voltage. Here, $\gamma _{3,4} = 0$ and the other parameters are listed below Fig. 2. The dashed and solid lines indicate positive and negative spin polarization, $s_{z} = \pm 1/2$, respectively.

The above results are generalization of previous findings for bilayer graphene (see, e.g., Ref. ^50^). In Figure 4 we show how the energy dispersion and Berry curvature change with the gate voltage (here defined in the energy units). By changing the gate voltage from -8 meV to +8 meV, one can observe swapping between the domination of exchange or spin-orbit coupling in the valence and conduction electronic states. For example, the spin polarizations of the conduction bands in the K and K’ points are identical for ${V=8\textrm{meV}}$, which suggests the dominant role of exchange interaction in the corresponding electronics states, whereas the spin polarizations of the valence bands are opposite in the K and K’ points, which indicates on the dominant role of spin-orbit interaction in the electronic states. This is, because the spin-orbit interaction does not break the time-reversal symmetry, and thus leads to the opposite spin splitting in the K and K’ points, whereas the spin splitting due to the exchange interaction (that leads to breaking of the time-reversal symmetry) should be the same in the K and K’ points. Accordingly, tuning the gate voltage leads to swapping of the character of spin splitting in the valence and conduction bands, i.e., to the swapping between spin-orbital and exchange dominated character of the electronic states at the fixed Fermi level. Another important feature of the ’ex-so-tic’ structure is the fact that the energy spectrum in the K and K’s points is substantially different, which opens the route towards valley-contrasting phenomena in these structures.

## Results and discussion

**Fig. 5:**
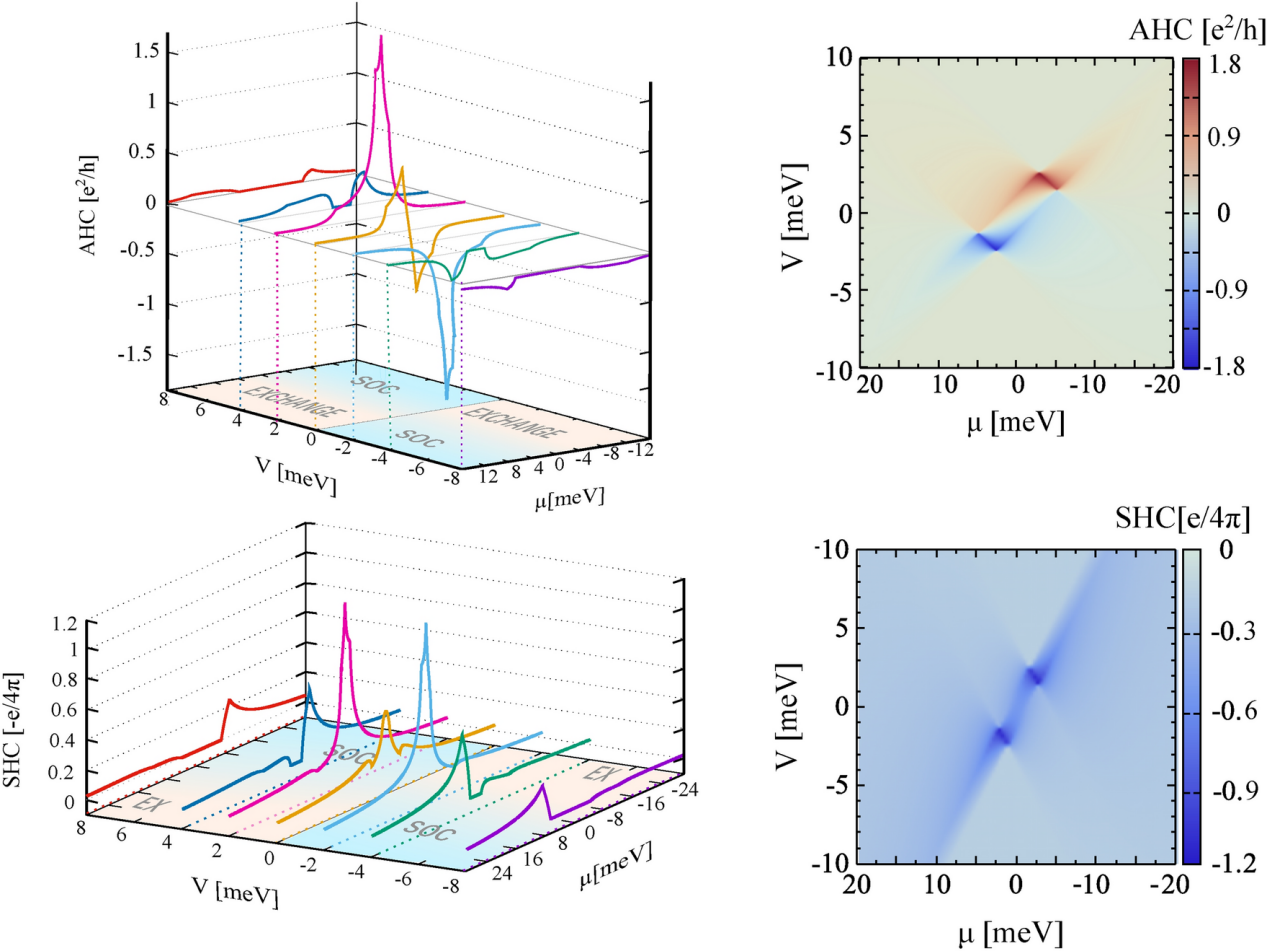
Anomalous Hall conductivity and spin Hall conductivity as a function of chemical potential, $\mu$, and gate voltage,*V*. The other parameters as in Fig. 2.

Here, we present and discuss the results obtained for the anomalous and spin Hall effects, as well as for two valley-contrasting phenomena known as the valley Hall effect and valley spin Hall effect. First, we present the results obtained when neglecting the interlayer hopping integrals $\gamma _{3,4}$. In such a case, all the numerical results presented in the next two subsections have been obtained based on fully analytical solutions (all formulas are presented in the supplementary material). Then, at the end we will discuss the influence of nonzero parameters $\gamma _{3,4}$.

### Anomalous and Spin Hall Effects

Figure 5 shows the anomalous Hall and spin Hall conductivities as a function of chemical potential and gate voltage. Both, anomalous and spin Hall conductivities do not achieve quantized values in the energy gap. Thus, there is no transition to a topologically nontrivial phase. The anomalous Hall conductivity displays very sharp peaks in a well-defined range of the chemical potential and gate voltage. More precisely, the anomalous Hall effect is negative for positive chemical potentials, $\mu$, and negative values of gate voltage, *V*, while it is positive for negative chemical potentials and positive values of gate voltage. Moreover, it displays very sharp peaks only in a very narrow range of $\mu$ and *V*. In the top right plot in Fig. 5, the peaks in the Hall conductivity are well-seen as the red and blue hot spots, that appear for the gate voltage range, where the spin-orbit interaction dominates in the valence or conduction bands. The peaks reflect the largest asymmetry in band structures and absolute values of Berry curvature in the vicinity of K and K’ points (see Fig. 4). In Fig. 5 one can also see, that the spin Hall conductivity behaves in a similar way. However, it is positive in the whole range of $\mu$ and *V*, and reveals peaks when the Fermi energy is in the valence or conduction band and the gate voltage ensure the dominant role of spin-orbit interaction in the corresponding electronic states. These characteristics of the anomalous and spin Hall effects can be very attractive in the context of applications and construction of electronic elements with very strong electronic signals only in a very specific energy range.

### Valley Hall effects

**Fig. 6:**
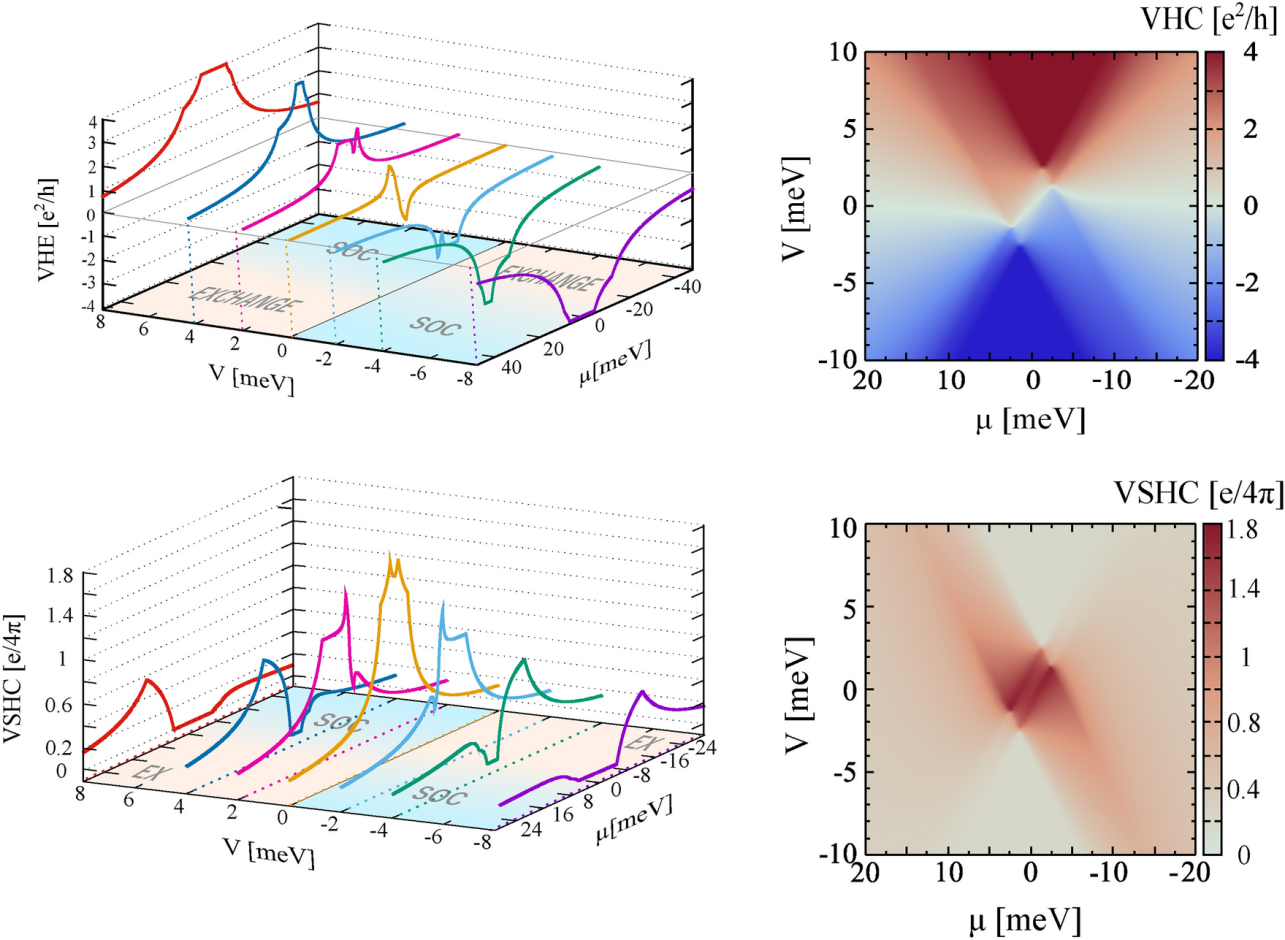
Valley Hall conductivity and valley spin Hall conductivity as a function of chemical potential, $\mu$, and gate voltage, *V*. The other parameters as in Fig. 2.

An important feature of the ’ex-so-tic’ structure under consideration is a clear distinction between the energy dispersion around the K and K’ points. This allows one to measure the system response that comes from only a single valley or at least is dominated by electronic states from a specific valley. Recently, the valley Hall effects in graphene-based systems have been considered in Refs^23,51,52^. However, as long as the energy bands are not distinguishable in the K and K’ points, the valley-dependent transport properties are very difficult to be measured. The ’ex-so-tic’ structure seems to be a big step forward in the development of valleytronics.

Figure 6 shows behaviour of the valley Hall and valley spin Hall conductivities as a function of the chemical potential and gate voltage. The valley Hall effect is nonzero in the system under consideration, and the valley Hall conductivity reaches the quantized value when the Fermi level is inside the energy gap. This quantized value changes from +4$\textrm{e}^{2}/h$ to $- 4\textrm{e}^{2}/h$, depending on the gate voltage. It is worth noting that the quantized valley Hall effect appears in the system because the Hall conductivity associated with electronic states in the K and K’ points achieves a quantized value equal $\pm 2\textrm{e}^{2}/h$ respectively for K and K’ points, when the Fermi level is inside the energy gap. This explains not only the quantized valley Hall conductivity, but also a zero anomalous Hall conductivity, when the Fermi level is in the energy gap. Figure 6 also shows, that in the ’ex-so-tic’ structures one can expect a nonzero valley spin Hall effect. The corresponding valley spin Hall conductivity, in contrast to the spin Hall conductivity, is positive for the whole range of parameters *V* and $\mu$. Moreover, the well-defined picks in the valley spin Hall conductivity are observed for the Fermi levels in the valence or conduction bands, depending on the value of gate voltage that ensures the dominant role of exchange interaction in electronic states.

### Effect of interlayer hopping $\gamma _{3}$ and $\gamma _{4}$

**Fig. 7:**
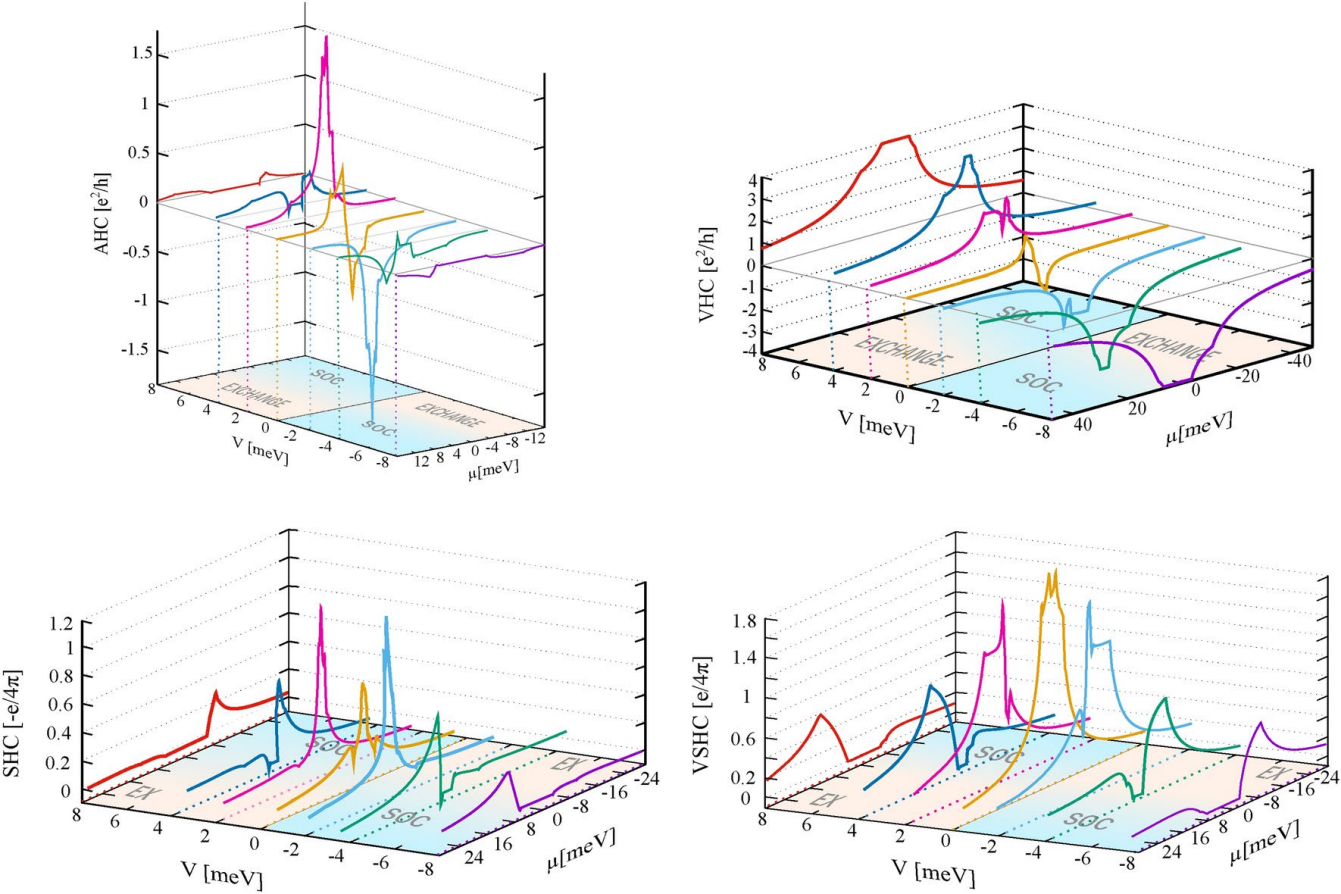
The anomalous, spin, valley and valley spin Hall conductivities as a function of chemical potential and gate voltage in the case of nonzero $\gamma _{3,4}$ hoppings. The parameters are as in Fig. 2.

**Fig. 8:**
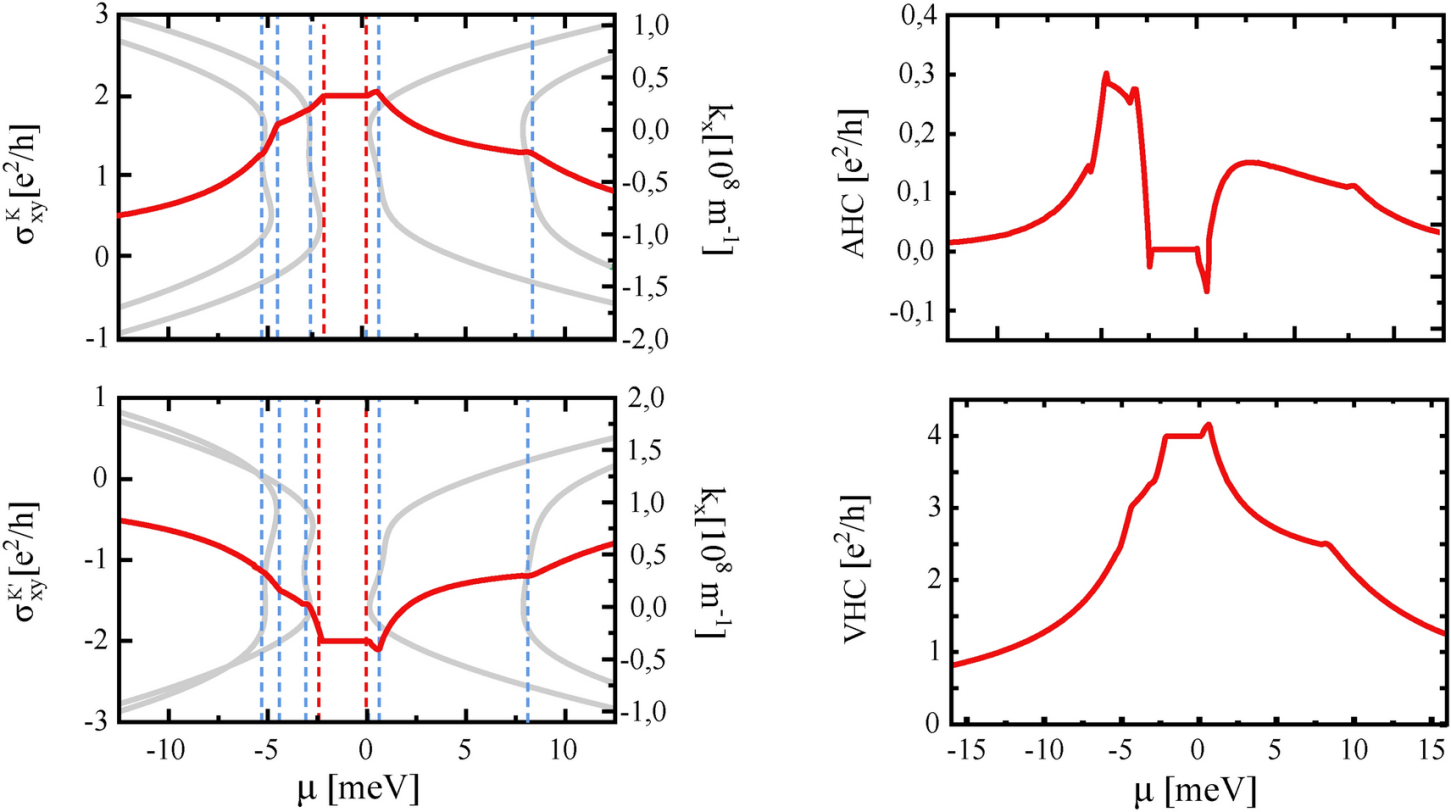
Charge Hall conductivity in K and K’ points (left panel) as a function of chemical potential, and anomalous and valley Hall effect (right panel) as a function of chemical potential in case of nonzero $\gamma _{3,4}$ hoppings. The parameters are as in Fig. 2.

Now, we briefly discuss the effect of nonzero hopping integrals $\gamma _{3,4}$. Fig. 7 presents all the Hall conductivities discussed above, but with $\gamma _{3,4}$ taken into account. The results have been obtained numerically and do not differ qualitatively from those presented in Figs. 5 and 6. However, in all these characteristics, one can identify additional kinks or spikes. These sharp spikes reflect the valley-contrasting physics and band structure. This is clearly visible in Fig. 8, where in the left panel we present the charge Hall conductivity for the K and K’ points as a function of chemical potential, and in the right panel their sum (i.e., the anomalous Hall conductivity) and their difference (i,e. the valley Hall conductivity). In this plot, it is clearly seen that each kink or spike in the Hall conductivity reflects the position of the local extremes in the energy bands.

## Conclusions

In this paper we have investigated the anomalous, spin, and valley-contrasting effects in the ex-so-tic van-der-Waals structure consisting of a bilayer graphene deposited on a ferromagnetic 2D insulator, such as ($$\hbox {Cr}_2$$$$\hbox {Ge}_2$$$$\hbox {Te}_6$$), and covered by a semiconducting 2D crystal, e.g., $$\hbox {WS}_2$$. Accordingly, one 2D crystal ensures proximity-induced exchange coupling, and the second one ensures proximity-induced spin-orbit coupling, and both affect the electronic structure of bilayer graphen. From the effective 8*x*8 kp-Hamiltonian of Zollner et al.^24^, we have derived the reduced Hamiltonian describing the low-energy spectrum of the ex-so-tic structure in the vicinity of charge neutrality point. Then, we used the Green’s function formalism to calculate the specific charge and spin Hall conductivities. Interestingly, for a fixed position of the Fermi level, one can tune the gate voltage to obtain strong nonzero anomalous or spin Hall conductivities. The well-picked anomalous Hall conductivity (or spin Hall conductivity), characteristic of the ex-so-tic structure can be useful for new elements for spintronics (such as diodes or transistors). Moreover, we have shown that the system can be tuned to the specific range of gate voltage and chemical potentials, for which it displays the quantized valley Hall conductivity, which can be equal $$\pm 4 \textrm{e}^{2}/h$$, depending on the sign of gate voltage.

## Methods

In this work the transverse dc electric and spin conductivity have been derived within the Green’s function formalism in the following valley-dependent form:

10 $$\begin{aligned} & \sigma _{xy}^{\tau } = \lim _{\omega \rightarrow 0} \frac{e^{2} \hbar }{\omega } \int \frac{d \varepsilon }{2\pi } \int \frac{d^{2}{\textbf{k}}}{(2\pi )^{2}} \textrm{Tr} \left[ {\hat{v}}_{x}^{\tau } G^{\tau }_{{\textbf{k}}}(\varepsilon + \omega ) {\hat{v}}_{y}^{\tau }G^{\tau }_{{\textbf{k}}}(\varepsilon ) \right] \end{aligned}$$

11 $$\begin{aligned} & \sigma _{xy}^{s_{z}\,\tau } = \lim _{\omega \rightarrow 0} \frac{e^{2} \hbar }{\omega } \int \frac{d \varepsilon }{2\pi } \int \frac{d^{2}{\textbf{k}}}{(2\pi )^{2}} \textrm{Tr} \left[ {\hat{j}}_{x}^{s_z\,\tau } G^{\tau }_{{\textbf{k}}}(\varepsilon + \omega ) {\hat{v}}_{y}^{\tau }G^{\tau }_{{\textbf{k}}}(\varepsilon ) \right] \end{aligned}$$

where ${\hat{v}}_{\alpha } = \frac{1}{\hbar } \frac{\partial {\hat{H}}_{\scriptscriptstyle {B1A2}}^{\tau }}{\partial k_{\alpha }}$ denotes the velocity operator ($\alpha = {x,y}$), and ${\hat{j}}_{x}^{s_z\,\tau } = \frac{1}{2}[{\hat{v}}_{x}^{\tau },{\hat{S}}_{z}]_{+}$ is the spin current density operator, with the spin operator defined as ${\hat{S}}_{z} = \frac{\hbar }{2}\hat{\eta }_{0} \otimes {\hat{s}}_z$. Furthermore, $G_{{\textbf{k}}}^{\tau }$ is the casual Green’s function defined as $G_{{\textbf{k}}}^{\tau } = [(\varepsilon + \mu + i\delta \textrm{sign}(\varepsilon ))\hat{\eta }_{0} \otimes {\hat{s}}_{0} - {\hat{H}}_{\scriptscriptstyle {B1A2}}^{\tau }]^{-1}$, where $\mu$ denotes the chemical potential, and $\delta \rightarrow 0^{+}$ (as we consider the clean limit). Taking into account contributions from both valleys (K and K’) one finds the following expressions for the anomalous and spin Hall conductivity: $\sigma _{xy}^{AH} = \sigma _{xy}^{K} + \sigma _{xy}^{K'}:=\textrm{AHC}$, $\sigma _{xy}^{SH} = \sigma _{xy}^{s_z\,K} + \sigma _{xy}^{s_z\,K'}:= \textrm{SHC}$, respectively.

In the clean limit, when we consider only topological contribution to the Hall conductivity, Eq. (10) can be rewritten in terms of the Berry curvature as follows:

12 $$\begin{aligned} \sigma _{xy}^{\tau } = \frac{e^{2}}{\hbar } \sum _j \int \frac{d^{2} {\textbf{k}}}{(2\pi )^{2}} \Omega _{j}^{\tau } f(E_{j}) \end{aligned}$$

where $f(E_{j})$ denotes the Fermi-Dirac distribution function for the j-th subband (for details see e.g.^23,53^).

To explore valley contrasting Hall effects, one needs to subtract the transverse charge (spin) Hall conductivity for the K and K’ points. Accordingly, we use the following definitions for valley $\sigma _{xy}^{VH} = \sigma _{xy}^{K} - \sigma _{xy}^{K'}:=\textrm{VHC}$ and valley spin Hall effects $\sigma _{xy}^{VSH} = \sigma _{xy}^{s_z\,K} - \sigma _{xy}^{s_z\,K'}:= \textrm{VSHC}$.

## Supplementary Information

Supplementary Information.

## Data Availability

All data that support the findings of this study are included within the article (and any supplementary files).
