# Supplementary material for: Intrinsic anomalous, spin and valley Hall effects in ’ex-so-tic’ van-der-Waals structures
Source: Sci Rep. 2024 Oct 11;14:23808. doi: 10.1038/s41598-024-74596-x (PMC11470101; doi:10.1038/s41598-024-74596-x)
Supplement: Supplementary file 1 — Supplementary Information. [file 41598_2024_74596_MOESM1_ESM.pdf]

Supplementary Material

Intrinsic anomalous, spin and valley Hall effects in ex-so-tic van-der-Waals structures

I. Wojciechowska and A. Dyrdał
Faculty of Physics, ISQI, Adam Mickiewicz University in Poznań, Poland
ul. Uniwersytetu Poznańskiego 2, 61-614 Poznań

1 Model

Here we present the full 8x8 $\mathbf{k}\cdot\mathbf{p}$ Hamiltonian describing ex-so-tic graphene-based structure as well as the derivation of reduced Hamiltonian presented in the main text and used in our calculations.

2 8x8 $\mathbf{k}\cdot\mathbf{p}$ Hamiltonian

The effective $\mathbf{k}\cdot\mathbf{p}$ Hamiltonian describing CGT/BLG/TMDC structure has been derived in Ref.[1] based on DFT modelling and symmetry considerations. This Hamiltonian describes electronic states in the vicinity of K and K' points (indexed by $\tau = \pm 1$) of the Brillouin zone[1]:

$$\hat{H}^{\tau} = \hat{H}^{\tau}_{ORB} + \hat{H}^{\tau}_{SOC} + \hat{H}^{\tau}_{R} + \hat{H}_{EX}. \tag{1}$$

The first term of the above Hamiltonian describes the orbital physics of the structure and takes the form[1]:

$$\begin{aligned}\hat{H}^{\tau}_{ORB} = &-\frac{\sqrt{3}\gamma_0 a}{2}\hat{\mu}_0 \otimes (\tau k_x \hat{\sigma}_x + k_y \hat{\sigma}_y) \otimes \hat{s}_0 + \frac{\gamma_1}{2}(\hat{\mu}_x \otimes \hat{\sigma}_x - \hat{\mu}_y \otimes \hat{\sigma}_y) \otimes \hat{s}_0 \\ &- \frac{\sqrt{3}\gamma_3 a}{4}\hat{\mu}_x \otimes (\tau k_x \hat{\sigma}_x - k_y \hat{\sigma}_y) \otimes \hat{s}_0 - \frac{\sqrt{3}\gamma_3 a}{4}\hat{\mu}_y \otimes (\tau k_x \hat{\sigma}_y + k_y \hat{\sigma}_x) \otimes \hat{s}_0 \\ &- \frac{\sqrt{3}\gamma_4 a}{2}(\tau k_x \hat{\mu}_x - k_y \hat{\mu}_y) \otimes \hat{\sigma}_0 \otimes \hat{s}_0 \\ &+ V \hat{\mu}_z \otimes \hat{\sigma}_0 \otimes \hat{s}_0 + \Delta(\hat{\mu}_+ \otimes \hat{\sigma}_+ + \hat{\mu}_- \otimes \hat{\sigma}_-) \otimes \hat{s}_0,\end{aligned} \tag{2}$$

where $\gamma_{0,1,3,4}$ define the intralayer electron hopping between nearest neighbors, as well as interlayer hoppings between nearest and next nearest sites, as indicated in Fig.1 in the main text, a is the lattice constant of graphene, V describes the effect of gate voltage (transverse displacement field), and Δ is the so-called orbital gap, being a consequence of the asymmetry in the energy shift of the bonding and antibonding states.
The next term describes the intrinsic spin-orbital proximity effect and has the following form[1]:

$$\hat{H}^{\tau}_{SOC} = \hat{\mu}_+ \otimes \tau(\lambda_I^{A1}\hat{\sigma}_+ + \lambda_I^{B1}\hat{\sigma}_-) \otimes \hat{s}_z - \hat{\mu}_- \otimes \tau(\lambda_I^{A2}\hat{\sigma}_+ + \lambda_I^{B2}\hat{\sigma}_-) \otimes \hat{s}_z, \tag{3}$$

with the spin-orbit coupling constant λ_I^{Xn}, where $X = \{A, B\}$ and $n = \{1, 2\}$ indicate sublattice (A or B) in the top (1) or bottom (2) layer, $\hat{\sigma}_\pm = (\hat{\sigma}_z \pm \hat{\sigma}_0)/2$, and $\hat{\mu}_\pm = (\hat{\mu}_z \pm \hat{\mu}_0)/2$.
The Rashba Hamiltonian for BLG reads[1]:

$$\hat{H}^{\tau}_{R} = \frac{1}{2}(\lambda_{IR}\hat{\mu}_z + 2\lambda_{BR}\hat{\mu}_0) \otimes (\tau\hat{\sigma}_x \otimes \hat{s}_y - \hat{\sigma}_y \otimes \hat{s}_x), \tag{4}$$

where λ_{IR} describes the strength of the so-called intrinsic Rashba spin-orbit coupling (or Dresselhaus-like SOC), originating from a local bulk-inversion-asymmetry due to the contact with adjacent layers; and λ_{BR} is the Bychkov-Rashba coupling constant due to the global space symmetry breaking[2].
The magnetic proximity effect responsible for the exchange interaction in BLG as a result of interaction with an adjacent magnetic layer with out-of-plane anisotropy (i.e., magnetization oriented in the z-direction) is described by the following term[1]:

$$\hat{H}_{EX} = \hat{\mu}_+ \otimes (-\lambda_{EX}^{A1}\hat{\sigma}_+ + \lambda_{EX}^{B1}\hat{\sigma}_-) \otimes \hat{s}_z - \hat{\mu}_- \otimes (-\lambda_{EX}^{A2}\hat{\sigma}_+ + \lambda_{EX}^{B2}\hat{\sigma}_-) \otimes \hat{s}_z \tag{5}$$

where λ_{EX}^{Xn} ($X = \{A, B\}$, $n = \{1, 2\}$) is the parameter describing the strength of proximity exchange coupling in the sublattice X of the n-th layer.

In the following considerations we assume $\lambda_I^{A2} = \lambda_I$, $\lambda_I^{B2} = -\lambda_I$, $\lambda_{EX}^{A1} = \lambda_{EX}^{B1} = \lambda_{EX}$

2.1 Reduced low-energy Hamiltonian

As the transport properties are related to the four low-energy bands (for each Dirac point) in the vicinity of the Fermi level, one can further reduce the 8×8 Hamiltonian (1) to a simpler 4×4 form. We derived the reduced Hamiltonian using the Green function method[3,4]. In the first step the Hamiltonian (1) was written in the basis (B1↑,B1↓,A2↑,A2↓, A1↑,A1↓,B2↑, B2↓) and separated into 2×2 blocks:

$$\hat{H}^{\tau} = \begin{pmatrix} \mathbb{H}_{11} & \mathbb{H}_{12} \\ \mathbb{H}_{21} & \mathbb{H}_{22} \end{pmatrix} \tag{6}$$

In consequence one can define the block $\mathbb{H}_{22}$ that is formed by A1-B2 dimer (related to the higher energy states), and the block $\mathbb{H}_{11}$ that is formed by low-energy states. Next, the Hamiltonian (6) has been expanded with respect to the parameter $p = 1/\gamma_1$. This procedure allows one effectively to exclude the atomic sites involved in the A1-B2 dimer bond. Accordingly, the Green function related to (6) has the form:

$$G = \begin{pmatrix} \mathbb{G}_{11} & \mathbb{G}_{12} \\ \mathbb{G}_{21} & \mathbb{G}_{22} \end{pmatrix}, \tag{7}$$

where G_{11} contains information on the low-energy states and can be used to determine the effective reduced Hamiltonian[3]. Using the definition of the Green function one can write

$$G = \begin{pmatrix} \mathbb{H}_{11}-\varepsilon & \mathbb{H}_{12} \\ \mathbb{H}_{21} & \mathbb{H}_{22}-\varepsilon \end{pmatrix}^{-1} = \begin{pmatrix} \mathbb{G}_{11}^{0-1} & \mathbb{H}_{12} \\ \mathbb{H}_{21} & \mathbb{G}_{22}^{0-1} \end{pmatrix}^{-1} \tag{8}$$

where

$$\mathbb{H}^{0}_{\alpha\alpha} = (\mathbb{H}^{0}_{\alpha\alpha} - \varepsilon)^{-1}. \tag{9}$$

Evaluation of (8) gives:

$$\mathbb{G}_{11}^{-1} + \varepsilon = \mathbb{H}_{11} - \mathbb{H}_{12}\mathbb{G}^{0}_{22}\mathbb{H}_{21}. \tag{10}$$

Assuming that $|\varepsilon| \ll \gamma_1$, the expression defining $\mathbb{G}^{0}_{22}$ can be expanded with respect to the parameter $p = 1/\gamma_1$. Finally, the reduced Hamiltonian corresponding to $\mathbb{G}_{11}$ can be written in the form:

$$\begin{aligned} \hat{H}^{\tau}_{B_1A_2} = & -\frac{v^2}{\gamma_1}\left(1+\gamma_{40}^2\right)\left((k_x^2-k_y^2)\hat{\eta}_x - 2k_xk_y\hat{\eta}_y\right)\otimes \hat{s}_0 \\ & -2\frac{v^2}{\gamma_1}\gamma_{40}k^2(\hat{\eta}_0\otimes\hat{s}_0) - v\gamma_{30}(\tau k_x\hat{\eta}_x + k_y\hat{\eta}_y)\otimes\hat{s}_0 \\ & -\lambda_{EX}(\hat{\eta}_+\otimes\hat{s}_z) + \lambda_I\tau(\hat{\eta}_-\otimes\hat{s}_z) + V(\hat{\eta}_z\otimes\hat{s}_0), \end{aligned} \tag{11}$$

where $\gamma_{30} = \gamma_3/\gamma_0$, $\gamma_{40} = \gamma_4/\gamma_0$, and $\hat{\eta}_\alpha$,$\hat{\eta}_0$ represent Pauli matrices and identity matrix acting in the B1-A2 dimer space, $k_\pm = k_x\tau \pm ik_y$, and $\hat{\eta}_\pm = \frac{1}{2}(\sigma_z \pm \sigma_0)$.

References

1. Zollner, K., Gmitra, M. & Fabian, J. Swapping Exchange and Spin-Orbit Coupling in 2D van der Waals Heterostructures. *Phys. Rev. Lett.* **125**, 196402, DOI: 10.1103/PhysRevLett.125.196402 (2020).
2. Konschuh, S., Gmitra, M., Kochan, D. & Fabian, J. Theory of spin-orbit coupling in bilayer graphene. *Phys. Rev. B* **85**, 115423, DOI: 10.1103/PhysRevB.85.115423 (2012).
3. McCann, E. & Fal'ko, V. I. Landau-Level Degeneracy and Quantum Hall Effect in a Graphite Bilayer. *Phys. Rev. Lett.* **96**, 086805, DOI: 10.1103/PhysRevLett.96.086805 (2006).
4. Abergel, D. & Apalkov et al, V. Properties of graphene: a theoretical perspective. *Adv. Phys.* **59**, 261 – 482, DOI: 10.1080/00018732.2010.487978 (2010). https://doi.org/10.1080/00018732.2010.48798.
